# A Marriage of Old and New: Chemostats and Microarrays Identify a New Model System for Ammonium Toxicity

**DOI:** 10.1371/journal.pbio.0040388

**Published:** 2006-11-14

**Authors:** Michael C Lorenz

## Abstract

Toxicity is related to an organism's ability to rid itself of the offending molecules. This primer provides insights into how this can be monitored by highlighting the case of ammonium toxicity.

Ammonium (NH_4_^+^), the substance created by the fixation of atmospheric nitrogen (N_2_), occupies a central role in cellular nitrogen metabolism. However, even though it is an important metabolite, free ammonium is present only transiently in biological systems, where it is rapidly converted into amino acids—initially glutamate and, particularly, glutamine. From there, transamination reactions convert it into other amino acids and nitrogen-containing cellular components.

The scarcity of free ammonium is not accidental. Ammonium and ammonia (NH_3_; the term ammonium is used here generically) are highly reactive compounds with substantial cytotoxicity. Ammonium detoxification is the primary function of the urea cycle in mammals, which converts the amine group from arginine into urea in the liver before it is excreted. Above-normal levels of ammonium (hyperammonemia), principally as a result of liver damage, can cause a variety of pathologies, the most serious of which is swelling of the brain, termed hepatic encephalopathy. In plants, high ammonium concentrations (in the low millimolar range) in the soil can retard growth, impair root development, and cause a yellowing of the leaves (chlorosis).

Ammonium toxicity in plants is an interesting and troubling problem (for a more thorough discussion, see [1]). Ammonium levels in soils are generally rising, presaging increasingly severe effects on plant life. The rising concentrations come from the heavy agricultural use of ammonium-based fertilizers; at low concentrations, ammonium is an ideal nutrient for plants. This clearly illustrates the paradox of ammonium: in multicellular organisms, it is a nutrient, a metabolite, and a toxin.

As for many other processes, a genetically tractable model system would help shed light on these contradictory roles. In microbes, however, ammonium is considered a preferred nitrogen source, one capable of supporting optimal growth rates even at high levels. In fact, Escherichia coli and Bacillus subtilis grow well in even molar concentrations of ammonium [2], making a bacterial model for ammonium toxicity unlikely.

## Ammonium Toxicity in Yeast

Similarly, the model eukaryote Saccharomyces cerevisiae avidly uses ammonium as a nutrient. Synthetic growth media commonly contain 76 mM ammonium sulfate (5 g/l), severalfold higher than the concentrations that inhibit the growth of most plants, and it has never been suggested that this “preferred” nitrogen source is harmful in any way. Thus, one might not expect the great similarities in cellular physiology between yeast and higher eukaryotes to extend to ammonium toxicity.

But in this issue, David Hess, Josh Rabinowitz, and David Botstein at Princeton's Lewis-Sigler Institute demonstrate for the first time that S. cerevisiae is indeed sensitive to ammonium [3]. Surprisingly, ammonium is toxic only when cells are deprived of potassium (K^+^). At 13 mM potassium, yeast thrive in ammonium concentrations of 600 mM and higher. At 1.3 mM potassium, however, there is significant growth retardation even at the “normal” level of 76 mM ammonium. Moreover, this is specific to ammonium—no potassium-related growth differences were noted when asparagine was used as an alternate nitrogen source (thanks to its nitrogen-rich side chain).

Just as important as these findings is the manner in which they were made. The appreciation of ammonium toxicity in yeast came through two departures from standard laboratory experimentation: the authors used physiologically relevant concentrations of the primary nutrient in question, and they used a growth reactor (a chemostat) that allowed them to control precisely the composition of the media at all stages of growth. Finally, their genomic approach, which is by no means unusual, shares with forward genetic screens the advantage of allowing the organism to share its secrets without having to make a priori assumptions.

## Neither Richer nor Poorer

The initial motivation for this study was to understand the physiology of potassium limitation; discovering the connection to ammonium toxicity was fortuitous and unexpected. The work follows highly informative investigations from this lab on yeast grown in conditions limited for phosphate, sulfur, amino acids, or nucleotides [4]. “Limitation” in these experiments does not mean absence. To understand the difference, it is useful to consider how nutrient starvation experiments are typically conducted.

Standard rich laboratory media is designed to support optimal growth, not to study nutrient homeostasis, and it contains an overabundance of key nutrients. The substantial body of literature on nutrient starvation in many systems has generally come from cells transferred from rich medium to the same medium entirely lacking a single compound. Alternatively, cells are grown for a period of time in the presence of a concentration of one nutrient that can be quickly exhausted. In nature, however, most organisms exist in conditions in which nutrients are present but not plentiful. Physiological responses are only informative if the conditions tested bear some similarity to the environmental stresses in which the organism evolved—and in general, that is not rich media. It could be argued that the lifespan extension seen under caloric restriction in yeast and in animals is the evolutionary result of these environmental pressures.

Like other nutrients, potassium is oversupplied in most yeast media: 10-fold or more above the concentration in seawater or human serum. Reducing the potassium level by 10-fold, as was done here, more closely resembles the natural environment and offers a more relevant view of potassium limitation.

## The Chemostat

The standard way to grow yeast (and most microbial cultures) in the laboratory is in an Erlenmeyer flask on an orbital shaker. The mixing and aeration afforded by this technique permit maximal growth rates. Cells stop growing when some essential nutrient is depleted, usually the carbon source in yeast cultures. When growth finally stops, one does not know the final composition of the medium—the cells have been using nutrients at different rates and have excreted waste products. Altering the beginning medium formulation (with less potassium, for instance) will then change, in unanticipated ways, the contents of the medium at the end of the experiment, and it illustrates a principal drawback of batch cultures—the inability to precisely control the environmental conditions.

To overcome this problem, Hess et al. turned to a chemostat. In this device, cells and spent medium are removed at a constant rate and replaced at the same rate with fresh medium. At steady state, the culture is diluted at the same rate as cell division—even though the cells are growing, the total number of cells in the chemostat does not change over time. Thanks to this constancy, the medium composition remains roughly unchanged as well. Under these conditions, specific changes to nutrient levels can be made while limiting unintended secondary effects. As a result, Hess et al. could be confident that the transcription changes in nitrogen metabolism they observed were indeed due to potassium limitation and not to other unintended variations. The importance of this experimental control was documented in the earlier work of the Botstein group, in which they concluded that the activation of the general stress response during nutrient starvation, as seen in other studies, was an artifact of the batch culture method [4].

The use of chemostats to carefully control environmental conditions was first described independently by Monod and by Novick and Szilard in 1950 [5,6]. Used extensively in the heyday of microbial physiology and biochemistry in the 1960s, chemostats then fell out of favor until a recent resurgence driven by data-heavy genomics and proteomics experiments, in which greater homogeneity in culture conditions improves both inter- and intra-lab reproducibility (discussed more completely in [7]). Reproducibility is a cornerstone of scientific research, and the immense size of these data sets makes it easier to quantify variance while at the same time making it much harder to reduce this variability.

Chemostats can be difficult to set up and optimize, and this effort is certainly not necessary in many types of experiments. But for those in which reproducibility and precise environmental control are important, they are a vital tool. A primer on chemostat design and use written by members of Maitreya Dunham's lab (Princeton University, Princeton, New Jersey, United States) is available at http://www.genomics.princeton.edu/dunham/MDchemostat.pdf.

## Let the Organism Do the Hard Work

The connection between ammonium and potassium homeostasis was unexpected. The authors did not make assumptions about what might occur during potassium limitation but simply designed the experiment in a manner that allowed the organism to reveal its secrets. Such projects are sometimes dismissed as not being “hypothesis-driven” or as “fishing expeditions,” because they do not test a specific model. But in this case, the underlying assumption was very basic: yeast cells must have specific responses to limited potassium availability. Identifying the processes and players first is a vital step in laying the foundation for detailed hypotheses and models.

The classical approach to studying this problem would have been to do a genetic screen for mutants that were either less able, or more able, to grow in limiting potassium media. Today, a variety of “-omics” approaches complement such forward genetic screens: genomics, proteomics, metabolomics. In this study, the authors started with transcript profiling. Although plenty of caveats accompany microarray studies, they are a reasonably unbiased means to identify genes, regardless of function, that respond to specific perturbations. With no preconceived notion about the results, the authors identified the long-overlooked connection between potassium limitation and ammonium metabolism.

## Ammonium Detoxification

**Figure 1:**
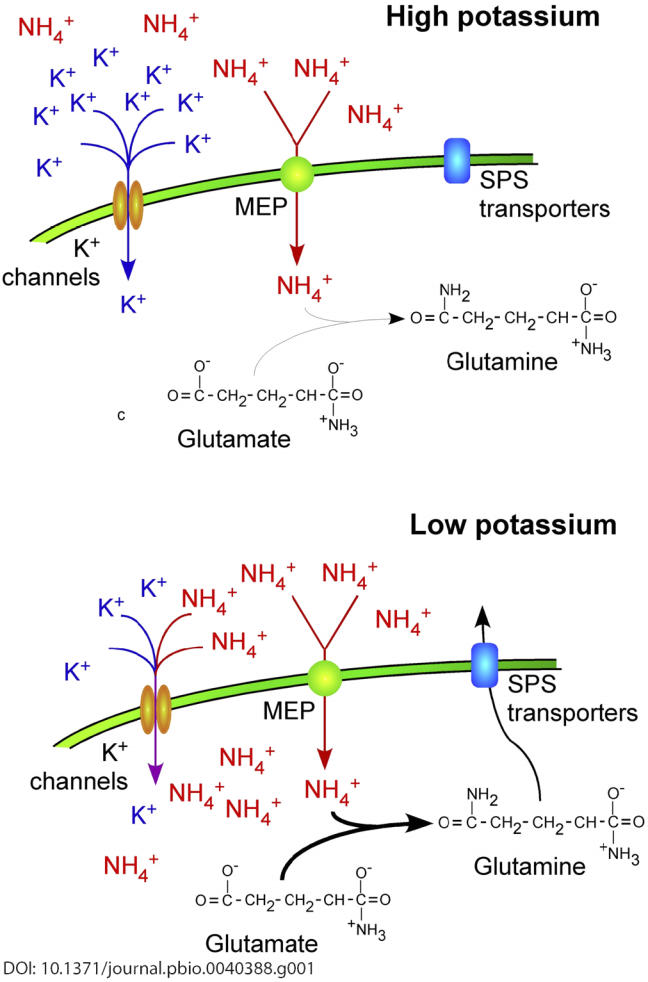
A Model of Ammonium Detoxification in Yeast In high-potassium conditions, ammonium enters the cell only via the MEP ammonium permeases. This low influx is rapidly converted into glutamine and, from there, into other nitrogen-containing compounds. When potassium is limiting, ammonium can also leak through potassium channels. To remove this unregulated inflow of ammonium, far more amino acids are produced than can be used for cellular functions. The remainder is exported via the bidirectional, SPS-regulated amino acid transporters.

To return to ammonium toxicity in yeast: an obvious question is how these two small ions are related. While not directly addressed by Hess et al., previous work has suggested that ammonium can be transported at low affinity via potassium channels in several systems (Figure 1). Thus, a reasonable hypothesis is that low external potassium levels do not completely occupy these channels, thereby allowing ammonium to leak in. Overexpression of the ammonium transporters MEP1, MEP2, and MEP3 (homologs of the bacterial AmtB and mammalian Rhesus proteins) also impaired growth independently of potassium concentration in the Hess et al. study, supporting this idea.

The microarray data also suggested how yeast detoxifies ammonium: through the export of amino acids. It has long been known that ammonium is rapidly converted into amino acids, but the revelation that this might be linked to export came from the microarray data. A large number of amino acid transporters were induced, most of which are regulated by the SPS system (named for the constituent proteins SSY1, PTR3, and SSY5) that controls the uptake of all amino acids other than arginine. These transporters are passive and therefore bidirectional, and although they primarily import amino acids into the cell, “leakage” of amino acids back into the media has been observed. Mass spectroscopy demonstrated the presence of large quantities of amino acids in the medium of potassium-limited chemostats. Because the starting medium did not contain amino acids, any free amino acids must have come from the cells themselves.

The export of amino acids distinguishes yeast somewhat from mammals, where excess nitrogen is converted into urea in the liver and eliminated. However, in humans with an impaired urea cycle due to liver damage from disease (cirrhosis, hepatitis), trauma, or transplantation, a back-up system functions to detoxify ammonium, which is converted into glutamine primarily in skeletal muscle and the brain. As a strong osmolyte, glutamine induces the brain-swelling characteristic of hepatic encephalopathy (for a review of the connection between ammonium, hepatic encephalopathy, and glutamine, see [8]). It is tempting to speculate that glutamine production is a vestige of the ancestral ammonium detoxifying system still found in yeast and that the urea cycle is an evolutionary adaptation in animals to overcome the effects of amino acid extrusion.

## Conclusions

The work from the Botstein group has shown that ammonium can have contradictory physiological roles in yeast, just as it does in plant and animal cells: it is the preferred nitrogen source for a yeast cell but can become toxic under certain conditions. Moreover, detoxification in yeast via amino acid release has evolutionary similarities with the mechanisms used by animals to eliminate ammonium. The tools available in the yeast system can now be used to address how free ammonium is deleterious to the cell and how amino acid synthesis as a detoxification mechanism is regulated.

Just as important as the specific findings, however, is the philosophy of this work. It is widely recognized that the laboratory conditions under which most model systems are grown bear little resemblance to the environment in which that species evolved. Just as artificial are the feast-or-famine approaches to understanding nutrient deprivation. The utility of studying conditions in which nutrients are scarce, but not absent, becomes obvious in this study. The chemostat growth reactor, allowing precise environmental control, combined with an unbiased assay system enables the investigators to dissect the physiological responses to nutrient limitation in a biologically meaningful manner. The Botstein group is certainly not the first to recognize this, but their accompanying paper [3] is strong evidence of the benefits of such approaches.
